# A systemmatic literature review on indirect costs of women with breast cancer

**DOI:** 10.1186/s12962-022-00408-6

**Published:** 2022-12-12

**Authors:** Saeed Mohammadpour, Samira Soleimanpour, Javad Javan-Noughabi, Nasrin Aboulhasanbeigi Gallehzan, Ali Aboutorabi, Reza Jahangiri, Rafat Bagherzadeh, Julia F. Gorman, Ali Nemati

**Affiliations:** 1 Department of Health Economics, School of Health Management and Information Sciences, Iran University of Medical Sciences, Tehran, Iran; 2 Department of Medical Library and Information Science, School of Health Management and Information Sciences, Iran University of Medical Sciences, Tehran, Iran; 3 Social Determinants of Health Research Center, Mashhad University of Medical Sciences, Mashhad, Iran; 4 Department of Health Economics and Management Sciences, School of Health, Mashhad University of Medical Sciences, Mashhad, Iran; 5 Department of Health Economics, Health Management and Economics Research Center, Iran University of Medical Sciences, Tehran, Iran; 6 English Language Department, School of Health Management and Information Sciences, Iran University of Medical Sciences, Tehran, Iran; 7 Graduate School of Humanities and Social Sciences, University of Melbourne, Melbourne, Australia; 8 Department of Health Services Management, School of Health Management and Information Sciences, Iran University of Medical Sciences, Tehran, Iran

**Keywords:** Breast neoplasms, Breast cancer, Indirect cost, Female, Systematic review

## Abstract

**Introduction:**

The rising incidence of breast cancer places a financial burden on national health services and economies. The objective of this review is to present a detailed analysis of the research and literature on indirect costs of breast cancer.

**Methods:**

English literature databases from 2000 to 2020 were searched to find studies related to the objective of the present review. Study selection and data extraction was undertaken independently by two authors. Also, quality assessment was done using a checklist designed by Stunhldreher et al.

**Results:**

The current study chose 33 studies that were eligible from a total of 2825 records obtained. The cost of lost productivity due to premature death based on human capital approach ranged from $22,386 to $52 billion. The cost burden from productivity lost due to premature death based on friction cost approach ranged from $1488.61 to $4,518,628.5. The cost burden from productivity lost due to morbidity with the human capital approach was reported as $126,857,360.69 to $596,659,071.28. The cost of lost productivity arising from informal caregivers with the human capital approach was $297,548.46 to $308 billion.

**Conclusion:**

Evaluation of the existing evidence revealed the indirect costs of breast cancer in women to be significantly high. This study did a thorough review on the indirect costs associated with breast cancer in women which could serve as a guide to help pick the appropriate method for calculating the indirect costs of breast cancer based on existing methods, approach and data. There is a need for calculations to be standardised since the heterogeneity of results in different domains from various studies makes it impossible for comparisons to be made among different countries.

## Introduction

Breast cancer has now surpassed lung cancer as the leading cause of global cancer incidence in 2020, with an estimated 2.3 million new cases, representing 11.7% of all cancer cases [1]. The global incidence of breast cancer in women is estimated to reach as many as 3.2 million new cases annually by the year 2050 [2]. Breast cancer is the fifth leading cause of cancer deaths worldwide and is estimated to have caused 684,996 deaths in 2020 [3]. Incidence rates for breast cancer far exceed those of other cancers in both transitioned (55.9 per 100,000) and transitioning (29.7 per 100,000) countries [1]. Breast cancer is considered the primary cause of woman mortality worldwide, accounting for 15% of total mortality among women [4]. According to the American Cancer Society, one in eight women experiences breast cancer during her life [2]. However, the prevalence of breast cancer in developed countries is higher than that in developing countries, also known as low- and middle-income countries (LMICs); nonetheless, the prevalence of breast cancer has recently been growing in LMICs as well [4].

The impact of this disease is clear not only in terms of mortality and morbidity but also in terms of economic consequences for all National Health Services (NHSs) and from a social point of view [5, 6]. The three cancers with the highest economic burden in the world are lung cancer ($188 billion), colon/rectal cancer ($99 billion), and breast cancer ($88 billion) [7]. Therefore, studies on the economic burden of diseases are valuable because of the rising costs of cancer diagnosis and treatment [8].

Ranganathan et al. suggested that due to the high economic burden of breast cancer in LMICs, the need to improve the management of patients with breast cancer in these countries is of great importance [9]. In this regard, various studies have been conducted on the economic burden of breast cancer in LMICs such as Iran [8, 10]. Also, studies have shown that different breast cancer treatments can impose different costs on society and patients [5, 6]. Cost of illness studies can be very helpful in determining the cost effectiveness of diagnosis and treatment of the disease and thus the optimal use of resources.

The cost of illness is investigated using a variety of methods [11]. From a social perspective, the cost of a disease consists of three main components: direct costs, indirect costs, and intangible costs [6]. Indirect costs are caused by the productivity loss resulting from disease or treatment side effects, which also affect patient’s family and those who care about them [6]. Indirect costs consist of two parts, temporary and permanent. The temporary indirect costs are the reduction of productivity due to disability and the permanent indirect costs are the loss of productivity due to mortality [12]. Indirect costs are an important component of costs of illness studies, especially in the management of chronic diseases that may require lifelong treatment [13]. In addition to medical and therapeutic expenses, women must shoulder the costs pertinent to missed work days or productivity costs in paid employment or at home [14–16]. Absenteeism can vary from a few weeks to several months. The risk of job loss among people diagnosed with cancer is 1.3 times higher than those without cancer [17]. Even when diagnosed at an early stage, breast cancer can adversely affect an individual’s ability to work for up to 5 years after the original diagnosis [18].

Factors associated with impaired productivity include adverse effects and treatments, such as progression and exacerbation of disease, cognitive and neurological disorders, poor physical and mental health, chemotherapy, and the time and cost required to receive treatment [19]. In Zheng et al.’s (2016) study, nonelderly women with breast cancer, compared with other people, significantly experienced job incapacity (13.6%), including reduced productivity at work (7.2 days) and at home (3.3 days) [20]. In another study, reduction in productivity due to adverse effects from breast cancer in the Netherlands and Sweden was 68% and 72% respectively [21]. Some patients may never return to work due to disability or premature death. Between 2012 and 2018, the lost productivity cost of premature deaths due to cancers in Iran has increased from $2453 million to $2887 million (An 18% increase) [22]. The issue of indirect costs is important in high income countries due to increasing prevalence rates of breast cancer in these countries [23]. Indirect costs are critical in LMICs not only because the growing burden of cancers in LMICs—but also issues of resources and affordability [4, 24, 25].

Despite the simplicity of expressing the components of indirect costs, the proper method of measuring and evaluating the productivity costs of breast cancer can be problematic. There are several methods to measure indirect costs [26]. The most accurate estimation of indirect costs requires the use of micro-costing methods; thus, it requires relatively large sample sizes, well-designed protocols, and well-trained interviewers [11, 27]. In an economic evaluation, the methods used for the measurement and evaluation of productivity costs can affect the results of the studies [26]. The use of different methods for calculating the productivity costs may impede the comparison of results between countries. Possible reasons for the differences in indirect costs include methodology, the value of local productivity, disease and patient characteristics, social security systems, and epidemiologic environments [28].

Therefore, the aim of this study was to systematically review the indirect costs and the monetary value of productivity costs due to breast cancer in women.

## Methods

This systematic review was conducted in compliance with the Preferred Reporting Items for Systematic Reviews and Meta-Analyses (PRISMA) guidelines [29].

### Design and eligibility criteria

We included published studies meeting the following criteria: (1) participants were female patients with breast cancer, (2) the outcome measures were related to indirect costs due to breast cancer. Cost of illness studies which included estimates of indirect costs of breast cancer at a municipal level (for example, city, state, country) or within certain organizations (for example, at employer level, or within health insurance companies) were also taken into account, (3) as for the design, only original articles were considered, and (4) only papers published in English were included in the study. Moreover, economic evaluation studies, reviews, letters, abstracts, conference papers, and general commentary or perspectives were excluded from the study.

### Search strategy

A systematic search was conducted in six electronic databases including PubMed, EMBASE, Scopus, Web of Science from 2000 until September 30, 2020.

Groups of keywords were chosen to search in selected databases without language restriction. Search strings were limited to the title, abstract and keywords. The keywords and scripts were developed using the US National Library of Medicine’s Medical Subject Headings (MeSH) and Emtree from Embase database. A complete search strategy for databases showed in Table 1.

**Table 1:** Search strategy

Search syntax	Database
(“Indirect cost”[TIAB] OR “Cost of illness”[MH] OR **“**Illness Cost”[TIAB] OR “Sickness Cost”[TIAB] OR “Burden of Illness”[TIAB] OR “Illness Burden”[TIAB] OR “Cost of Disease”[TIAB] OR “Economic Burden of Disease”[TIAB] OR “Disease Cost”[TIAB] OR “Disease Costs”[TIAB] OR “Cost of Sickness”[TIAB] OR “Sickness Costs”[TIAB] OR “Costs of Disease”[TIAB] OR “Productivity costs”[TIAB] OR “Productivity lost”[TIAB] OR “Productivity loss”[TIAB] OR “Absenteeism cost”[TIAB] OR “Human capital”[TIAB] OR “Economic burden”[TIAB]) AND ("Breast Neoplasms"[MH] OR "Breast Tumors"[TIAB] OR "Breast Tumor"[TIAB] OR "Breast Carcinoma"[TIAB] OR "Breast Cancer"[TIAB] OR "Mammary Cancer"[TIAB] OR "Mammary Cancers"[TIAB] OR "Malignant Neoplasm of Breast"[TIAB] OR "Breast Malignant Neoplasm"[TIAB] OR "Breast Malignant Neoplasms"[TIAB] OR "Malignant Tumor of Breast"[TIAB] OR "Breast Malignant Tumor"[TIAB] OR "Breast Malignant Tumors"[TIAB] OR "Cancer of Breast"[TIAB] OR "Cancer of the Breast"[TIAB] OR "advanced breast cancer"[TIAB] OR "mamma cancer"[TIAB] OR "mammary gland cancer"[TIAB])	Pubmed
TS=(“Indirect cost” OR “Cost of illness” OR **“**Illness Cost” OR “Sickness Cost” OR “Burden of Illness” OR “Illness Burden” OR “Cost of Disease” OR “Economic Burden of Disease” OR “Disease Cost” OR “Disease Costs” OR “Cost of Sickness” OR “Sickness Costs” OR “Costs of Disease” OR “Productivity costs” OR “Productivity lost” OR “Productivity loss” OR “Absenteeism cost” OR “Human capital” OR “Economic burden”) AND **TS=**("Breast Neoplasms" OR "Breast Tumors" OR "Breast Tumor" OR "Breast Carcinoma" OR "Breast Cancer" OR "Mammary Cancer" OR "Mammary Cancers" OR "Malignant Neoplasm of Breast” OR "Breast Malignant Neoplasm" OR "Breast Malignant Neoplasms" OR "Malignant Tumor of Breast" OR "Breast Malignant Tumor" OR "Breast Malignant Tumors" OR "Cancer of Breast" OR "Cancer of the Breast" OR "advanced breast cancer" OR "mamma cancer” OR "mammary gland cancer")	Web of Science
TITLE-ABS-KEY (“Indirect cost” OR “Cost of illness” OR **“**Illness Cost” OR “Sickness Cost” OR “Burden of Illness” OR “Illness Burden” OR “Cost of Disease” OR “Economic Burden of Disease” OR “Disease Cost” OR “Disease Costs” OR “Cost of Sickness” OR “Sickness Costs” OR “Costs of Disease” OR “Productivity costs” OR “Productivity lost” OR “Productivity loss” OR “Absenteeism cost” OR “Human capital” OR “Economic burden”) AND TITLE-ABS-KEY ("Breast Neoplasms" OR "Breast Tumors" OR "Breast Tumor" OR "Breast Carcinoma" OR "Breast Cancer" OR "Mammary Cancer" OR "Mammary Cancers" OR "Malignant Neoplasm of Breast” OR "Breast Malignant Neoplasm" OR "Breast Malignant Neoplasms" OR "Malignant Tumor of Breast" OR "Breast Malignant Tumor" OR "Breast Malignant Tumors" OR "Cancer of Breast" OR "Cancer of the Breast" OR "advanced breast cancer" OR "mamma cancer” OR "mammary gland cancer")	Scopus
(“Indirect cost”/exp “Indirect cost”:ti,ab,kw OR “Cost of illness”:ti,ab,kw OR **“**Illness Cost”:ti,ab,kw OR “Sickness Cost”:ti,ab,kw OR “Burden of Illness”:ti,ab,kw OR “Illness Burden”:ti,ab,kw OR “Cost of Disease”:ti,ab,kw OR “Economic Burden of Disease”:ti,ab,kw OR “Disease Cost”:ti,ab,kw OR “Disease Costs”:ti,ab,kw OR “Cost of Sickness”:ti,ab,kw OR “Sickness Costs”*:ti,ab,kw* OR “Costs of Disease”:ti,ab,kw OR “Productivity costs”:ti,ab,kw OR “Productivity lost”:ti,ab,kw OR “Productivity loss”:ti,ab,kw OR “Absenteeism cost”:ti,ab,kw OR “Human capital”:ti,ab,kw OR “Economic burden”:ti,ab,kw) AND ("Breast Cancer"/exp OR "Breast Cancer":ti,ab,kw OR "Breast Neoplasms":ti,ab,kw OR "Breast Tumors":ti,ab,kw OR "Breast Tumor":ti,ab,kw OR "Breast Carcinoma":ti,ab,kw OR "Mammary Cancer":ti,ab,kw OR "Mammary Cancers":ti,ab,kw OR "Malignant Neoplasm of Breast":ti,ab,kw OR "Breast Malignant Neoplasm":ti,ab,kw OR "Breast Malignant Neoplasms":ti,ab,kw OR "Malignant Tumor of Breast":ti,ab,kw OR "Breast Malignant Tumor":ti,ab,kw OR "Breast Malignant Tumors":ti,ab,kw OR "Cancer of Breast":ti,ab,kw OR "Cancer of the Breast":ti,ab,kw OR "advanced breast cancer":ti,ab,kw OR "mamma cancer":ti,ab,kw OR "mammary gland cancer":ti,ab,kw)	Embase

### Quality assessment

The qualitative analysis was carried out by two researchers (SM and NAG) using a checklist designed by Stunhldreher et al. [30]. The results of this analysis was scrutinized by the third researcher (SS). The following items were assessed: scope, general economic characteristics, and calculation of costs, study design and analysis, and presentation of the results.

### Selection of studies and data extraction

The list of publications generated from the literature search were entered into EndNote V.X8.1, and subsequent duplicates were removed. For controlling the validity of the study, two researchers (S.M and R.J) independently screened each retrieved record by reviewing the title and abstract. After that, two researchers (S.M and S.S) independently screened the full texts of the selected publications to match the eligibility criteria. The results of this analysis was scrutinized by the third researcher (J.J.N). The researchers reported the characteristics of the included studies, and a summary table of various checklists was completed to inform the assessments of the methodological quality of the cost of illness studies. The following details were extracted for each included study: first author’s name, publication year, reference year for cost, region, sample size, methodology of the study, costing approach, components of indirect costs, and estimated indirect costs. Also, in order to make comparisons among different studies, all costs were converted into US dollars based on the purchasing power parity index in 2020. Moreover, if an article did not mention the year for which the expenses were assumed, the year of publication of the article was considered the basis for the cost adjustment.

## Results

### Study selection

**Fig. 1:**
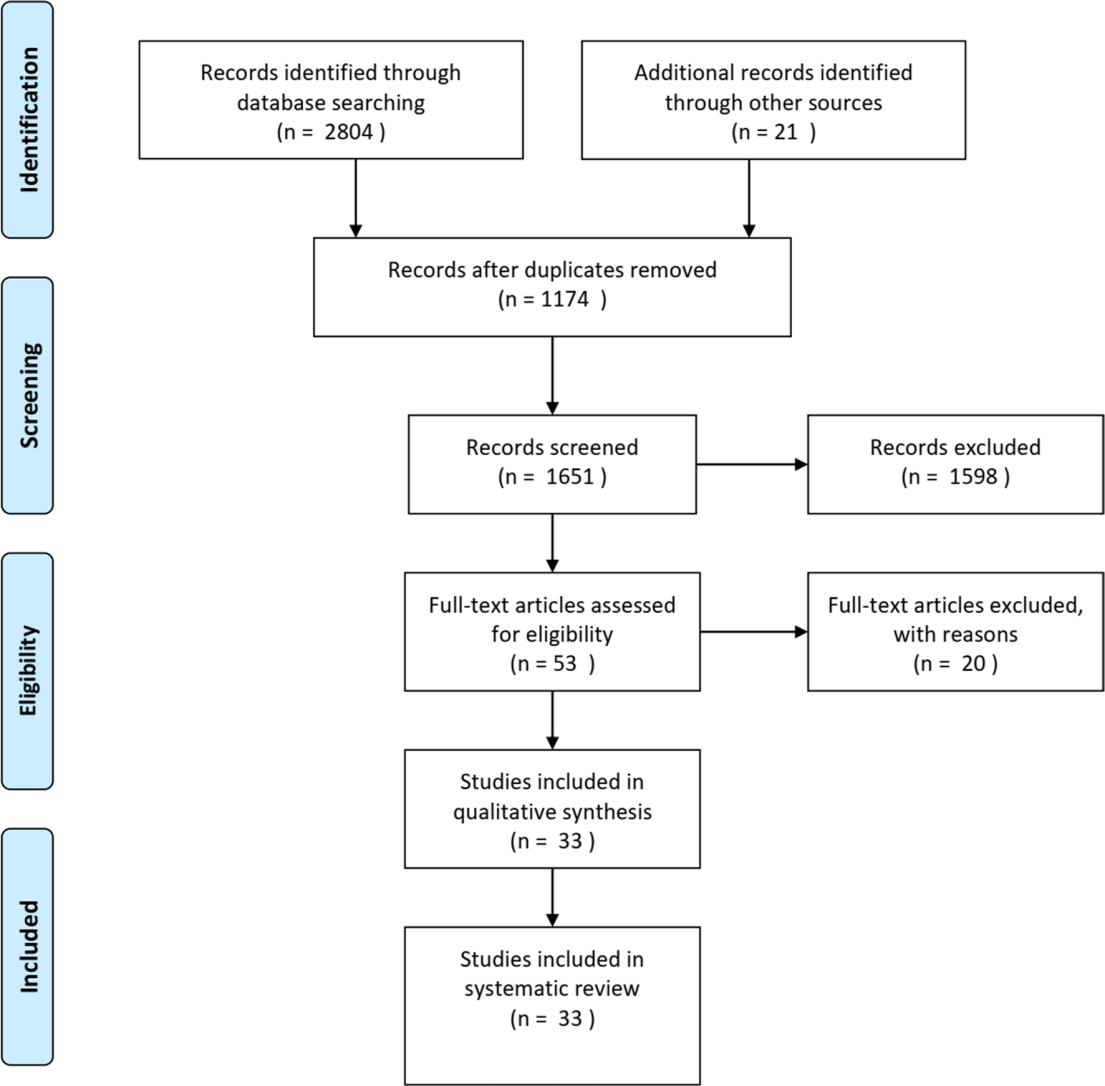
Study selection process

As a result of searching the target databases, 2825 relevant items were retrieved using the search queries. After discarding 1174 duplicate items, the number of items was reduced to 1651 items. We selected 53 records for full-text screening to reach the eligibility for analysis. finally, 33 articles were included based on the inclusion and exclusion criteria. Figure 1 shows the steps of searching and selecting papers.

### Quality assessment

The quality of the all 33 studies were evaluated based on the cost of illness (COI) tool, and all studies were considered appropriate to be included in the review. More details about quality assessment are reported in Table 2.

**Table 2:** Quality assessment of included studies

Criteria/author	Scope			General economic		Calculation of costs						
	Study objective	Inclusion and exclusion criteria	Disease and diagnostic criteria	Cost-description	Nondiseased comparison group or disease-specific costs	Currency	Reference year	Perspective	Costs incorporated from more than one category	Data source	Valuation of costs	Discounting
Lidgren [45]	✓	✓	✓	✓	✗	✓	✓	✓	✓	✓	✓	✗
Ivanauskiene [40]	✓	✓	✓	✓	✗	✗	✓	✓	✓	✓	✓	✗
Broekx [38]	✓	✓	✓	✓	✓	✗	✗	✓	✓	✓	✓	✓
Jain [55]	✓	✗	✗	✓	✗	✗	✗	✗	✗	✓	✓	✗
Łyszczarz [35]	✓	✗	✗	✓	✗	✓	✓	✓	✓	✓	✓	✗
Vondeling [52]	✓	✗	✓	✓	✗	✓	✓	✗	✓	✓	✓	✗
ROINE [58]	✓	✓	✓	✓	✓	✓	✓	✗	✓	✓	✓	✗
Trogdon [51]	✓	✗	✓	✓	✗	✗	✓	✗	✓	✓	✓	✗
Max [46]	✓	✗	✗	✓	✗	✗	✗	✗	✓	✓	✓	✓
Meadows [57]	✓	✗	✗	✓	✓	✗	✓	✓	✗	✓	✓	✗
Sorensen [50]	✓	✗	✓	✓	✗	✓	✗	✗	✓	✓	✓	✗
Heras [60]	✓	✗	✓	✓	✗	✓	✓	✗	✓	✓	✓	✓
Gordon [54]	✓	✓	✓	✓	✗	✓	✓	✗	✓	✓	✓	✗
Wan [31]	✓	✓	✓	✓	✓	✗	✗	✗	✓	✓	✓	✗
Binazzi [53]	✓	✓	✓	✓	✗	✓	✓	✗	✗	✓	✓	✗
Mahmood [56]	✓	✓	✓	✓	✗	✓	✓	✗	✓	✓	✓	✗
Goyal [61]	✓	✓	✓	✓	✗	✓	✓	✗	✓	✓	✓	✗
Ferrier [32]	✓	✓	✓	✓	✗	✗	✗	✗	✗	✓	✗	✗
Daroudi [10]	✓	✗	✓	✓	✗	✓	✓	✗	✓	✓	✓	✗
Yin [59]	✓	✓	✓	✓	✗	✓	✓	✗	✓	✓	✓	✗
Oliva [47]	✓	✗	✗	✓	✗	✓	✗	✗	✓	✓	✓	✓
Hanly [39]	✓	✗	✓	✓	✗	✓	✓	✓	✓	✓	✓	✓
Kim [43]	✓	✗	✓	✓	✗	✓	✓	✓	✓	✓	✓	✓
Pearce [48]	✓	✗	✓	✓	✗	✓	✓	✗	✓	✓	✓	✓
Bradley [33]	✓	✗	✓	✓	✗	✓	✗	✓	✓	✓	✓	✓
Hanly [36]	✓	✗	✓	✓	✗	✗	✓	✗	✓	✓	✓	✓
Lee [44]	✓	✗	✓	✓	✗	✗	✗	✗	✓	✓	✓	✓
Khorasani [42]	✓		✓	✓	✗	✓	✓	✗	✓	✓	✓	✓
Karami-matin [41]	✓	✗	✗	✓	✗	✗	✗	✗	✗	✓	✓	✓
Sasser [49]	✓	✓	✓	✓	✓	✓	✓	✗	✓	✓	✓	✓
Luengo [34]	✓	✗	✓	✓	✗	✓	✓	✓	✓	✓	✓	✓
John [62]	✓	✗	✗	✓	✗	✓	✓	✗	✗	✓	✓	✗
Barchuk [37]	✓	✗	✓	✓	✗	✓	✓	✗	✓	✓	✓	✓

### Study characteristics

**Fig. 2:**
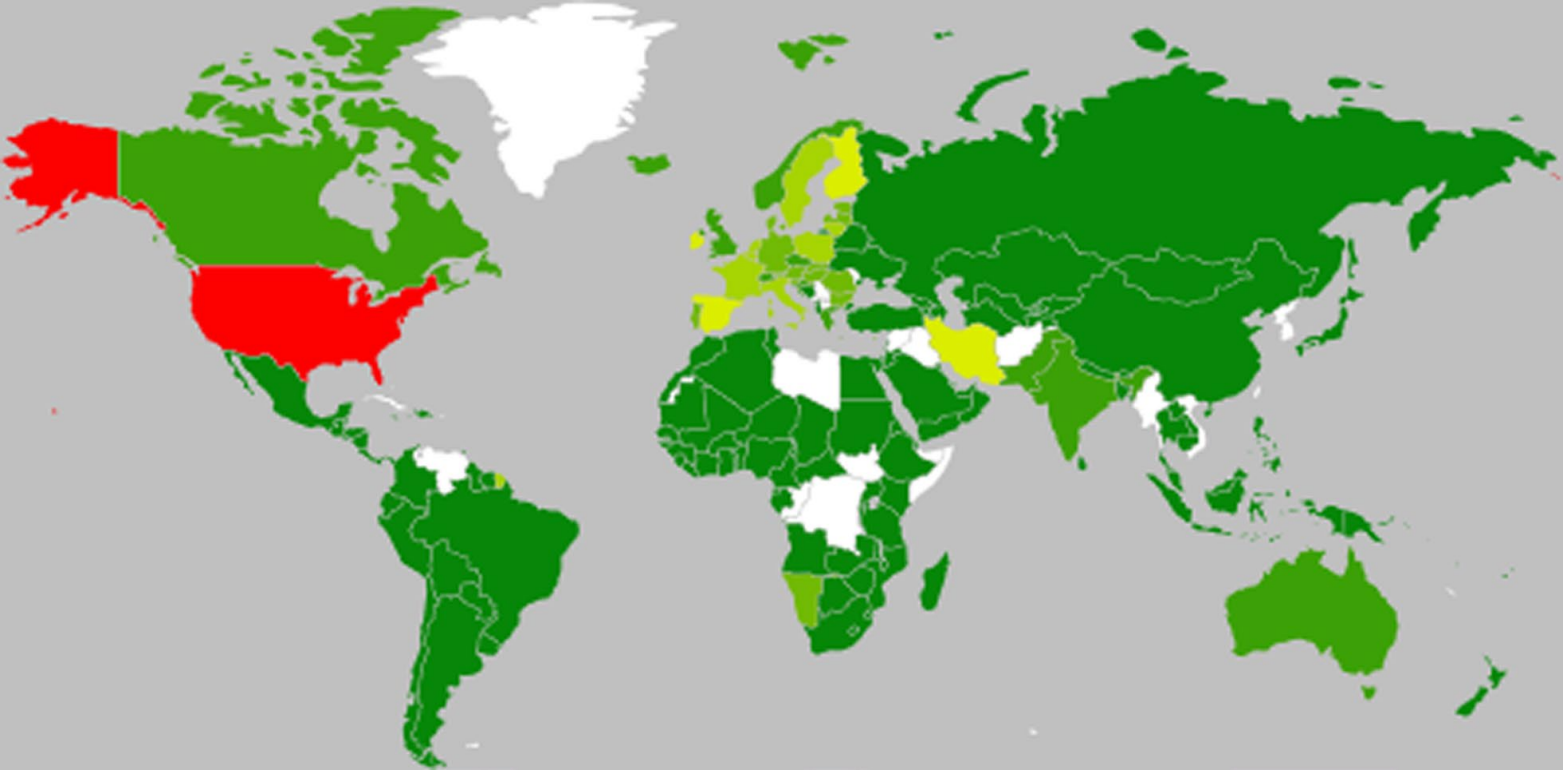
Regional distribution

Study characteristics of included articles shown in Fig. 2 and Table 3. According to research, the first study of indirect costs in breast cancer was published in 2005. The date of studies ranged from 2005 to 2020. The majority of the studies (n = 5) were conducted in 2018. The increasing number of publications over the last years shows that indirect costs have been an interesting topic in breast cancer research studies.

**Table 3:** Study charactristics of included articles

No.	First author	Study population	Databases	Perspective	Type of study	Discount rate	Region	Income groups	Sample size
1	Lidgren (2007) [45]	Female patients with a previous diagnosis of breast cancer	Enrolled	Societal	Prospective	–	Sweden	High income: OECD	361
2	Ivanauskiene (2010) [40]	A survey of 379 women treated in five major Lithuanian hospitals	Questionnaire	Societal	Prospective	–	Lithuania	High income: nonOECD	379
3	Broekx (2011) [38]	All women had undergone an initial surgical treatment for breast cancer between 1998 and 2003, allowing us to identify these patients in the Christian Health Insurance Funds databases based on the official billing codes attached to these surgical procedures	Enrolled	Societal	Prospective	4%	Belgium	High income: OECD	20,439
4	Jain (2016) [55]	The patients with primary diagnosis as breast cancer, diagnosed in between April 2012 to March 2013, not having any co-morbidities	Interview	Houshold	Prospective	–	India	Lower middle income	221
5	Łyszczarz (2017) [35]	Population based	Social insurance system and Polish National Cancer Registry	Societal	Retrospective	0%, 3.5%	Poland	High income: OECD	–
6	Vondeling (2018) [52]	Women with breast cancer in netherlands	Dutch National Cancer Registry	–	Retrospective	2%, 6%	Netherlands	High income: OECD	320,179
7	Roine (2019) [58]	All patients aged 18 years and over and diagnosed with BC were eligible for the study	questionnair	–	Prospective	–	Finland	High income: OECD	827
8	Trogdon (2020) [51]	women with no missing responses to questions regarding having ever been diagnosed with cancer, having ever been diagnosed with breast cancer, and age at breast cancer diagnosis	National Health Interview Survey	Social	Retrospective	3%	United States	High income: OECD	6935
9	Max (2009) [46]	California women for 2001 using California specific hospitalization and mortality data	California specific hospitalization and mortality data	–	Retrospective	3%	United States	High income: OECD	12,934
10	Meadows (2010) [57]	employed women, aged 18 to 64, with BC identified by a validated algorithm between 1999 and 2005, from claims (Market Scan) and attendance databases	Encounters (CC&E) and Health and Productivity Management (HPM) databases from Thomson Reuters	Employer	Reteospective	–	United States	High income: OECD	880
11	Sorensen (2012) [50]	The incident cohort of MBC patients included both de novo MBC patients and MBC patients who progressed during that year from earlier stages of breast cancer	Medical record	Social	–	3%	United States	High income: OECD	49,674
12	Heras (2018) [60]	Patients with newly diagnosed or recurrent mBC diagnosed over 1 year	Physician survey conducted with 10 clinical experts in Spain				Spain	High income: OECD	2923
13	Gordon (2007) [54]	English-speaking women recently diagnosed with unilateral breast cancer, aged 20–75 years and who resided within a 100 km radius of Brisbane (where approximately 70% of the Queensland population resides)	Questionnaire	The perspective of the survivor	Prospective	–	Australia	High income: OECD	287
14	Wan (2013) [31]	Adult BC patients eligible for employee benefits of sick leave and/or short-term disability were identified with ICD-9 codes	The MarketScan_ Health and Productivity Management database	societal	Retrospective	–	United State	High income: OECD	326,903
15	Binazzi (2013) [53]	Only subjects over 25 years deceased in 2006 have been selected by cancer site	Italian National Institute of Statistics	–	Retrospective	1%, 3%	Italy	High income: OECD	11,476 death
16	Mahmood (2018) [56]	Patients were eligible for inclusion if they were (1) female; (2) 18 years of age or older; (3) had been in treatment for 3 months to 2 years since diagnosis; (4) were diagnosed with metastatic breast cancer with any stage; (5) fluent in Urdu, English or regional languages i.e. Punjabi and Saraiki; and (6) able to provide informed consent	Qeustionnair	–	Prospective	–	Pakistan	Lower middle income	200
17	Goyal (2020) [61]	Patients with MBC was ascertained based on the presence of at least two claims with an ICD-9-CM diagnosis code for secondary malignancy	The IBM MarketScan Commercial Claims and Encounters (CCAE) databases	–	Retrospective	–	United State	High income: OECD	5563
18	Ferrier (2020) [32]	Female patients with histologically confirmed, previously untreated and primarily operable BC (exclusion of metastatic, locally advanced or inflammatory BC as defined by the AJCC)	Questionnaire	Socetial	Prospective		France	High income: OECD	168
19	Daroudi (2015) [10]	Cancer population	National cancer registry reports, hospital records, occupational data, and interviews with expert				Iran	Upper middle income	39,316
20	Yin (2017) [59]	The study sample included employees who had at least two inpatient or outpatient claims with a diagnosis of BC	The MarketScan_ Health and Productivity Management database	Employer	Retrospective	–	United State	High income: OECD	6409
21	Oliva (2006) [47]		Spanish Registry of Deaths by cause	–	Retrospective	0%, 3%, 6%	Spain	High income: OECD	38,025
22	Hanly (2012) [39]	Population-based sample of 1373 survivors was selected from the National Cancer Registry Ireland. Survivors were between 6 months and 2 years since diagnosis and had been treated at 1 of 17 hospitals across the country (14 mixed public/ private, 3 private)	Questionnaire	Societal (HCA) and an employer’s (FCA) perspective	Prospective	4%	Irland	High income: OECD	250
23	KIM (2007) [43]	All cancer population	Health Insurance Review agency & Korean Central Cancer Registry (KCCR)	Social	Retrospective	3%	Korea	High income: OECD	36,226
24	Pearce (2016) [48]	Cancer population	Central Statistics Office (CSO) and annual age-specific cancer	–	Retrospective & prospective	5%	Ireland	High income: OECD	Cancer death 2011–2030
25	Bradley (2008) [33]	All cancer population	National Interim Projections, Berkeley Mortality Database, Current Population Survey (CPS)	–	Retrospective	3%	United State	High income: OECD	–
26	Hanly (2014) [36]	Cancer deaths	WHO mortality database	Social	Retrospective	3.5%	Europe	–	–
27	Lee (2014) [44]	Women with breast cancer	national health insurance claims data	Societal	Retrospective	3%	Korea	High income: OECD	42,605 in 2000, 97,507 in 2010
28	Khorasani (2015) [42]	Cancer population	Iranian Ministry of Cooperation Labor and Social Welfare	Social	Retrospective	3%	Iran	Upper middle income	3304
29	Karami-matin (2016) [41]	Cancer people	Ministry of Health and Medical Education (MoHME) & Iranian Ministry of Cooperation Labor and Social Welfare	–	Retrospective	3%	Iran	Upper middle income	962 in 2006, 1,086 in 2007, 1,122 in 2008, 1,124 in 2009, 1,283 in 2010
30	Sasser (2005) [49]	Female employees, also age 50–64 years (the “comparison group”), during the 3-year period	Medical record	Employer	Retrospective	–	United State	High income: OECD	555
31	Luengo (2013) [34]	Population based	International and national sources		–	–	European Union	–	–
32	John (2010) [62]	Cancer population	WHO	–	Retrospective	–	Global	–	–
33	Barchuk (2019) [37]	Cancer population	Herzen Research Institute of Oncology	Societal burden of cancer in Russia	Retrospective	0% & 5%	Russia	High income: nonOECD	2031

The regional distribution of the studies shows that the research was undertaken involving 185 different countries.Based on World Bank Clasification and country income groups, most of the studies were conducted in high-income countries. As shown in Table 3 and Fig. 2, regions with red colour have the most number of studies. Countries with light green have more than one studies, and the ones with dark green have just one study. Furthermore, regions with white colour have no studies about indirect costs of breast cancer.

While studies have been conducted in different regions, one study was global and investigated all members of WHO countries. In addition, two studies were in European Union and investigated this Union’s countries. Regardless of these three studies, United States (n = 9) and Iran (n = 4) had the majority of studies in indirect costs of breast cancer.

The study findings displayed heterogeneity to varying degrees. The studies differed in terms of sample size, methods used, costing approach, study perspective, cost calculation, and data report per patient and per death. However, all studies reported indirect costs and cost productivity costs.

Regarding the study sample, the biggest sample size (326,903 people) was related to the study of Wan et al. [31]. And the smallest sample size (168 people) was related to the study of Ferrier et al. [32]. Five studies did not mention a specific sample size [7, 33–36].

### Items of indirect costs

Estimating the various items of indirect costs among women with breast cancer showed in Table 4. The results of our study showed that permanent indirect costs (due to mortality) estimated in 20 articles [10, 33, 35–52], productivity costs due to morbidity estimated in 21 studies [10, 31, 32, 35, 38–40, 43–45, 47, 49, 50, 52–59], and productivity costs due to informal caregivers (unpaid help) estimated in 8 articles [33–35, 50, 54–56, 58]. Also, there are no classification of indirect costs in 3 articles and this articles reported total indirect cost [60–62].

**Table 4:** Indirect costs among women with breast cancer

No.	First author	Reference year for costs	Region	Costing approach	Data gathering	Type of indirect cost	Cost (US dollars)
1	Mathias Lidgren [45]	2005	Sweden	HC	Not specified	Premature death	165,695.42
						Missed days’ work	20,167.36 to 48,407.67
						Total	33,992.96 for women aged lower than 50 years
							24,724.97 for women aged 50–64 years
2	Rugile Ivanauskiene [40]	2008	lithuania	HC	Not specified	Premature death	38,314,351.03
						Morbidity	69,856,613.68
						Total	150,204,061.57
3	Steven Broekx [38]	2006	Belgium	HC	Not specified	Premature death	33,930.20 per patient
						Morbidity	12,537.91 per patient
						Total	51,325.88
4	Maneeta Jain [55]		India	HC	Not specified	Missed days’ work	128,104.58
						Unpaid	297,548.46
						Total	1,337,388.34
						Productivity loss	14,014,584.40
5	Błażej Łyszczarz [35]	2010–2014	Poland	HC	Not specified	Premature death	103,782,672.49
						Morbidity	126,857,360.69
						Missed days’ work	79,153,502.28
						Unpaid help	301,578.48
						Total	434,722,812.46
6	G. T. Vondeling [52]	1990–2014	Netherlands	HC	Not specified	Premature death	331,729,844.30
						Morbidity	354,937,281.96
7	Eija Roine [58]	2009–2010	Finland	HC	Not specified	Mean loss productivity loss in primary treatment	11,743.73
						Mean loss productivity loss in metatatic	9794.59
						Sick leave	11,219.12
						Informal care	Primary treatment = 2895.30
							Metas = 3944.53
8	Justin G. Trogdon [51]	2015	United State	HC	Not specified	The value of lost work and home productivity days associated with mBC nationally	Younger women = 73,331,141.63
							Midlife women = 269,245,684.18
							Older women = 72,236,646.98
9	Wendy Max [46]	2001	United State	–	Prevalence base	Premature death	2,157,428,410.62
10	Eric S.Meadows [57]	2005	United State	–	Incidence based	Morbidity	6428.50
11	Sonja V.Sorensen [50]	2010	United State	–	Incidence based	Premature death	321,957,234.86
						Missed days’ work	301,934,425.64
						Unpaid help	54,773,302.74
						Total	682,998,152.02
12	de las.Heras [60]	2016	Spain	–	Incidence based	Total	388.41
13	Louisa Gordon [54]	2005	Australia	–	Not specified	Missed days’ work	2494$
						Unpaid help	435.56$
						Total	3732.47
14	Yin Wan [31]	Not specified	United State	–	Not specified	Morbidity	MBC = 6165.8
							EBC = 3689.7
						Missed days’ work	MBC = 1584
							EBC = 1015
15	Alessandra Binazzi [53]	2006	Italy	–	Not specified	Value of work productivity lost	71,767,637.28$
16	Hafiz Zahid Mahmood [56]	2015	Pakistan	–	Not specified	Missed days’ work	70.49
						Unpaid help	21.61
						Total	326.23
17	Ravi K.Goyal [61]	2015	United State	–	Not specified	Total	11,379.46
18	Clement Ferrier [32]	Not specified	France	HC&FC	Not specified	Missed days’ work	22,898.12(HC)
							Per patient
							7571.43(FC)
							Per patient
						Total	25,162.79(HC)
							Per patient
							8553.71(FC)
							Per patient
19	Rajabali Daroudi [10]	2010	Iran	HC	Prevalence base	Premature death	226,544.05
						Missed days’ work	6348.27
20	Wesley Yin [59]	2013	United State	HC&FC	Not specified	Missed days’ work	Non-metastatic: 27,238.20 metastatic: 34,564.53
21	Juan Oli va [47]	Not specified	Spain	HC&FC	Not specified	Premature death	223,328.35 based on HCA and 4,518,628.55 based on FCA
						Permanent disability	314,671,039.50 based on HCA and 10,771,684.06 based on FCA
						Total	570,359,693.86 based on HCA and 22,956,005.13 based on FCA
22	Paul Hanly [39]	2008	Ireland	HC&FC	Not specified	Premature death	108,419.60 based on HCA and 1488.61 based on FCA
						Morbidity	139,799.76 based on HCA and 8909.85 based on FCA
						Missed days’ work	63,566.23 based on HCA and 33.37 based on FCA
						Total	248,219.35 based on HCA and 10,398.46 based on FCA
23	KIM S.G [43]	2002	Korea	HC	Prevalence base	Premature death	253,290,344.47
						Morbidity	179,708,791.89
24	Alison Pearce [48]	2011–2030	Ireland	HC	Incidence based	Premature death	52,251,513,523.53
						Value of lost paid production	1,772,314,850.60
25	Cathy J. Bradley [33]	2010	United State	HC	Not specified	Premature death	12,981,917,165.92
						Unpaid help	308 billion
26	Paul Hanly [36]	2008		HC	Not specified	Premature death	5,742,690,674.97
27	Kwang-Sig Lee [44]	2010	Korea	HC	Not specified	Premature death	715,990,885.54
						Morbidity	596,659,071.28
28	Soheila Khorasani [42]	2012	Iran	HC	Not specified	Premature death	171,960,573.41
29	Behzad Karami-matin [41]	2006–2010	Iran	HC	Not specified	Premature death	5698.01 in 2006, 6088.31 in 2007, 5777.21 in 2008, 5900.88 in 2009, 4546.44 in 2010
30	Alicia C.Sasser [49]	1998–2000	United State	–	Not specified	Premature death	6760.01
						Morbidity	5338.09
31	Ramon Luengo [34]	2009	European Union	–	Not specified	Unpaid help	2,612,459,857.54
						Total	2,653,279,542.82
32	Rijo M John [62]	2008	Global	–	Not specified	Indirect cost	88 billions
33	Anton Barchuk [37]	2016	Russia	HC	Incidence based	Premature death	22,386

A further evaluation of the retrieved study showed that 17 studies employed the human capital approach (HCA) [10, 33, 35–38, 40–45, 48, 51, 52, 55, 58], four studies used both HCA and the friction cost approach (FCA) [32, 39, 47, 59]. Costing approach for estimating indirect cost was unclear in other studies [31, 34, 46, 49, 50, 53, 54, 56, 57, 60–62]. The productivity costs due to premature death with the HCA ranged from $22,386 to $52 billion. The productivity costs burden due to premature death with the FCA ranged from $1488.61 to $4,518,628.5. Some studies reported cost on the basis of per patient. Total indirect costs was $25,162.79 per patient based on HCA and $8553.71 per patient based on FCA. The productivity costs burden due to morbidity with the HCA was reported to be from $126,857,360.69 to $596,659,071.28. The productivity costs arising from informal caregivers with the HCA ranged from $297,548.46 to $308 billion. The productivity costs due to premature death with the HCA ranged from $22,386 to $52 billion. The productivity costs due to premature death with the FCA ranged from $1488.61 to $4,518,628.5. The productivity costs due to missed working days with the HCA ranged from $6,348.27 to $128,104.58. The productivity costs due to morbidity with the HCA varied from $126,857,360.69 to $596,659,071.28. The productivity costs from informal caregivers with the HCA ranged from $297,548.46 to $308 billion.

The current study found that the HCA was more commonly used to calculate indirect costs. This method derives the monetary value deterred from the productivity costs due to disability or premature death based on a person's wage prior to disability or death. This is considered an easy approach employed by many studies owing to the ease of access to the required data. The drawback of this approach is that it only considers the work that gets paid and does not include costs associated with home care and responsibilities outside the work place, like the case of housewives.

## Discussion

We conducted a systematic review of recently published studies on the indirect costs of breast cancer with the goal of identifying those whose methodological similarities would allow us to make comparisons and draw conclusions from the indirect cost burden of the disease [63]. According to evidence, cancer patients and their companions face high indirect costs. Indirect costs of cancer are the monetary losses associated with time spent receiving medical care, time lost from work or other usual activities (morbidity costs), and productivity costs due to premature death (mortality costs) [14].

The evaluation of the studies showed that most studies have calculated the productivity costs due to premature death, which can be because of easier access to the mortality data, while more data are needed to calculate other costs. The cost burden imposed by the productivity costs from premature death contributes profoundly to indirect costs and decreases productivity/efficiency. This number, as reported, ranges from $22,386 to $52 billion with the HCA and ranges from $1488.61 to $4,518,628.5 with the FCA. Moreover, housewives, too, get diagnosed with breast cancer alongside working women, more often resulting in early death, further contributing to the premature death rate. The cost of informal care, which involves non-monetary assistance provided by those around the cancer patient, was also witnessed to be high. With the HCA, this amount ranged from $297,548.46 to $308 billion. This is because of the fact that basically a cancer patient needs to be supported by family or friends either to be accompanied to the treatment centre or to receive high quality treatment at home. This forces the accompanying person to take time off to serve the patient.

The current study found that the HCA was more commonly used to calculate indirect costs. This method calculates the monetary value deterred from the productivity costs due to disability or premature death based on a person's wage prior to disability or death. This is considered an easy approach employed by many studies owing to the ease of access to the required data. The drawback of this approach is that it only considers the work that gets paid and does not include costs associated with home care and responsibilities outside the work place as with the case of housewives.

The study noted that five studies used the incidence approach and three studies used the prevalence approach. The incidence approach only examined new disease cases, but the prevalence approach examined both new patients and patients from the previous years. Unfortunately, the heterogeneity of the studies made it impossible to combine the results together to provide a single output in this field. Thus, this necessitates more research in this area. When evaluating indirect costs, the study perspective should be either focused on patients or communities. In addition, the community perspective tends to be the one widely used in the studies.

The magnitude of variability can be explained by the finding that different indirect cost elements are evaluated. Moreover, different methodologies for evaluating the same cost elements were used, and country differences can also provide reasoning to some extent [64]. Various methods were used to collect the data. These include questionnaire-based face-to-face interviews [40], telephone interviews [15], national study data [48–50], and even international data [35, 36].

Comparing indirect costs between the studies revealed in the systematic review is very difficult because of the possibility of the application of two different methods: the HCA and the FCA [65]. The results generated with these methods cannot be compared since the HCA estimates potential productivity costs, whereas the FCA presents the real value of it; the results achieved with these two methods are not comparable [66]. It should be noted that the HCA estimates costs more than the FCA. When assessing the indirect cost calculation methods, the willingness-to-pay (WTP) methodology did not appear in the studies included in this review.

Due to the methodological heterogeneity of the studies included in the review, focus was given to the qualitative analysis [64]. Thus, a great variety of methods and indirect cost components were shown in the studies, and it was impossible to carry out a meta-analysis. This review showed that further research is needed due to the lack of information on the topic, and a precise methodology of indirect cost estimation must be developed [65].

It appears that since breast cancer did not cause morbidity to the extent of, the DALY index (the disability-adjusted life year) was not used except in two studies [35, 58]. The high cost burden of mortality is reflected in the loss of active labour in society. Regardless of whether a woman is employed or not, women are traditionally responsible for domestic production; thus, premature mortality reduces domestic production. On the other hand, although gender wage gap exists where women are usually, on average, paid lower than men, there is still no evidence of lower productivity of women from various studies [39]. The world mathematician community may better be able to understand the impact of indirect costs of breast cancer after the death of Maryam Mirzakhani from breast cancer.

The great variety of indirect costs resulted from different cost components and macroeconomic indicators that were used for estimation purposes [65]. Economic results are difficult to compare on account of monetary issues, such as fluctuating exchange rates and different purchasing powers of currencies. Domestic characteristics also dramatically affect resource consumption and unit costs, including differences in clinical practice and the health-care system framework [67]. Using many macroeconomic characteristics for the purpose of indirect cost estimation is one of the major reasons for the significant variety of costs [68].

With regards to indirect costs, studies usually considered the average wage or per capita as a representative of lost earnings on working days. However, wage rate in developing countries such as Iran may be lower than optimal/average due to low labour productivity, but this is not expressed in the indirect costs.

Methods used to estimate disease costs vary widely across studies in the literature, which is probably due to the lack of consensus on the methodology. Few studies have been carried out around the world with a control group [33, 50]. Conducting case–control studies on national and international levels to calculate indirect costs associated with breast cancer is not a practical idea.

However, it should be added that comparing the costs between a group of patients with a disease and others that match in terms of confounding variables lends a better understanding of the cost burden of the disease. A study in Iran showed that the covid-19 pandemic caused an average of 16.44 absenteeism days and cost of $671.4 per patient [69]. It should also be kept in mind that almost all the studies carried out considered the natural history of disease in diagnosis and treatment. After the outbreak of the Covid-19, studies have been conducted on the changes in the course of the disease and the delay in the diagnosis and treatment of breast cancer, which has also affected the indirect costs of the disease [70–72].

A review of the studies showed that the researchers did not focus on foreseeing the indirect costs of this disease, and only one study foresaw the indirect costs of breast cancer [48]. Therefore, the definition of standards and consensus in the methodology selected to conduct these studies should be major concerns for the scientific community [67]. The review was limited to articles written in English, and we excluded non-English articles. Different studies accounted for different indirect cost elements and even used different methodologies for quantification.

## Conclusion

The current review provides important evidence of indirect costs associated with breast cancer which enables the economic burden of breast cancer to be predicted. Evaluating and measuring indirect costs give a better grasp of the reasons for the decline in productivity, for instance, informal assistance from those around the patient to the patient, the productivity costs from missed work days, the cost resulting from premature death and the damping cost elicited through the analysis of indirect costs. Women today make up a large portion of the labour force, while still in some communities they are responsible for household chores; hence, more focus on the disease often associated with women could provide more insight on the disease burden and its impact on the economy. While the findings of this study could be used to distinguish indirect and direct costs of breast cancer, it will also be helpful in economic evaluations of different treatment methods for this disease. Furthermore, it is necessary to mention the study approach (whether calculations were based on prevalence or incidence) to designate the appropriate sample size. Our study found that the indirect costs of breast cancer add highly to the cost burden of the disease. Also, the heterogeneity of various study results does not allow a consensus to be reached; therefore, it is imperative to standardise calculations, and since most of the studies have been conducted in high income countries, policy makers of the healthcare in middle and low income countries must prioritise research of such contexts.

### Recommendation

We suggest that more studies estimate the indirect costs of breast cancer in low- and middle-income countries as well. Also, our data on the costs related to breast cancer can be used to facilitate more economic evaluations.

### Limitation

Unfortunately, the present study did face some limitations. For instance, the heterogeneity of studies—the study population, geographical location, and calculation methods—were all diverse. Our study only reviewed studies that were published in English and excluded studies done in other languages. Studies of grey literature were also not included.

## Data Availability

Not applicable.
